# A runoff prediction method based on hyperparameter optimisation of a kernel extreme learning machine with multi-step decomposition

**DOI:** 10.1038/s41598-023-46682-z

**Published:** 2023-11-07

**Authors:** Xianqi Zhang, Fang Liu, Qiuwen Yin, Yu Qi, Shifeng Sun

**Affiliations:** 1 Water Conservancy College, North China University of Water Resources and Electric Power, Zhengzhou, 450046 China; 2 Collaborative Innovation Center of Water Resources Efficient Utilization and Protection Engineering, Zhengzhou, 450046 China; 3 Technology Research Center of Water Conservancy and Marine Traffic Engineering, Zhengzhou, 450046 Henan Province China

**Keywords:** Environmental sciences, Hydrology

## Abstract

To improve the accuracy of runoff forecasting, a combined forecasting model is established by using the kernel extreme learning machine (KELM) algorithm optimised by the butterfly optimisation algorithm (BOA), combined with the variational modal decomposition method (VMD) and the complementary ensemble empirical modal decomposition method (CEEMD), for the measured daily runoff sequences at Jiehetan and Huayuankou stations and Gaochun and Lijin stations. The results show that the combined model VMD-CEEMD-BOA-KELM predicts the best. The average absolute errors are 30.02, 23.72, 25.75, 29.37, and the root mean square errors are 20.53 m^3^/s, 18.79 m^3^/s, 18.66 m^3^/s, and 21.87 m^3^/s, the decision coefficients are all above 90 percent, respectively, and the Nash efficiency coefficients are all more than 90%, from the above it can be seen that the method has better results in runoff time series prediction.

## Introduction

The results of the medium- and long-term prediction of runoff are an important basis for rational scheduling, scientific planning, and comprehensive use of water resources, and also play an important role in the optimal operation of reservoirs, which is directly related to the industrial and agricultural production in the watershed and the development of the local socio-economy^1^. Therefore, it is of great practical significance to predict the change in runoff. The traditional methods of runoff prediction include hydrological modeling^2^ and statistical methods. There are generally problems of not easy access to parameters unsatisfactory fitting predictions, and complex model construction. In recent years neural networks^3^, grey prediction models^4^, regression models^5^ , and other intelligent methods have been gradually promoted and applied. The development of machine learning has provided new ideas for the prediction of complex runoff, in which the traditional shallow machine learning methods can successfully carry out the prediction of complex and non-stationary runoff, but their accuracy still needs to be further improved^6^. As artificial intelligence technology continues to evolve, deep learning is being introduced into the field of prediction. Among them, the Kernel Extreme Learning Machine (KELM) model has strong nonlinear forecasting ability, fast convergence speed and the ability to capture long-term correlation of time series, which can retain historical useful information for a long period of time^7^.

In the actual prediction process, due to the complexity of the runoff changes, a single prediction model will lose the important information implied in the original sequence, the prediction results and the actual runoff are difficult to fit well, so its coupled prediction model is getting more and more attention. In search of ways to improve the accuracy of predictions, Qiao et al.^8^ proposed a meta-heuristic evolutionary deep learning model based on Time Convolutional Network (TCN), Improved Aqua Hawk Optimiser (IAO) and Random Forest (RF) for rainfall runoff simulation and multi-step runoff prediction. RF is first used to calculate the correlation between the input variables and the predicted objects, and then the filtered data is sent to the TCN model. The parameters of the TCN model were optimised using the IAO algorithm, and the runoff from the Panzhihua site was simulated and predicted by building several models, and the results showed that the proposed model had the highest accuracy. A prediction model combining an integrated empirical modal decomposition (EEMD) method and a long short-term memory (LSTM) network was proposed by Huang et al.^9^. Combination of EEMD and K-means algorithms to decompose and reconstruct rainfall as the main variable affecting runoff into new sequences with greater regularity. The results show that the EEMD-LSTM multivariate model has better simulation performance than other models The EEMD-LSTM multivariate model is suitable for simulation and prediction of daily-scale rainfall-runoff processes in the rice area of southern China. An interval prediction method for monthly runoff based on WOA-VMD-LSTM was proposed by Wang et al.^10^. Variational Modal Decomposition (VMD) optimised using the Whale Optimisation Algorithm (WOA), followed by prediction of each subsequence using Long and Short Term Memory Neural Networks (LSTMs) to obtain the final point prediction. The results show that the predictive accuracy of the model is significantly higher than the other models used. Lian^11^ proposes a combined runoff prediction model based on complementary integrated empirical modal decomposition. The runoff data of Manas River in China is selected as the research object, and an improved fireworks algorithm is proposed to optimise the parameters of GPR and SVM models. Comparing the proposed combined model with the existing prediction model, the comparison result curves between the predicted and actual values of runoff, prediction errors, histograms of prediction error distribution, performance indexes and related statistical indexes show that the established prediction model has higher prediction accuracy and can correctly reflect the change rule of runoff. Zhang et al.^12^ constructed a coupled model based on MEEMD-ARIMA and applied it to the downstream runoff prediction of the Yellow River. The results show that the model has higher accuracy than the CEEMD-ARIMA model or EEMD-ARIMA model, and provides new ideas and methods for annual runoff prediction. Yan et al.^13^ proposed a model based on weighted integrated modified complementary integrated empirical modal decomposition to predict the monthly runoff at the lower Yellow River hydrological station. Particle swarm optimisation was used to optimise the parameters of the support vector regression, back-propagation neural network, and long- and short-term memory neural networks that make up the model. The weighting coefficients and frequency terms of the MCEEMD decomposition were used to obtain the final predictions. The results show that the model outperforms other models, with all error indicators minimised. Kernel extreme learning machines can improve the robustness of extreme learning machines by converting linearly non-separable data in low-dimensional spaces into linearly separable data. Lu et al.^14^ used the Algorithm for Particle Swimming Optimisation with Active Operators (APSO) to construct the optimal KELM classifier for APSO-KELM. Experiments show that APSO-KELM has higher classification accuracy than existing KELM models and algorithms combining PSO/APSO with ELM/KELM. Song et al.^15^ proposed a water quality assessment model based on the sparrow search algorithm optimised kernel extreme learning machine (KELM) applied to the Luoyang River Basin, where the extreme learning machine (ELM), KELM, support vector regression (SVR), and back-propagation neural network (BPNN) were used as baseline models to validate the proposed hybrid model. The results show that the water quality evaluation model based on KELM optimisation is superior to other models.

All of the models constructed by the above methods performed only single-step predictions and did not take into account the high complexity of the models. In this paper, different decomposition methods are used for multi-step decomposition prediction, and sample entropy is introduced to reorganise the components with similar complexity, reduce the number of components and decrease the time complexity of the model. For the prediction of daily runoff, this paper uses the kernel-limit learning machine algorithm developed on the basis of statistical learning theory. Statistical learning theory is a theory specialised in studying the laws of machine learning in the case of small samples, providing a unified framework for solving finite sample learning problems. It can incorporate many existing methods, which can help to solve many original difficult problems such as neural network structure selection problems, local extreme value problem, etc., and finally get the global optimal solution. Therefore, the kernel extreme learning machine (KELM) algorithm is adopted in this paper for prediction, while the butterfly optimisation algorithm is used to optimise the KELM model to get better prediction results, that is, the combined VMD-CEEMD-BOA-KELM prediction model is established and applied in runoff prediction of the Jiehetan, Huayuankou, Gaocun and Lijin stations.

## Research methodology and theory

### VMD-CEEMD decomposition algorithm

Variational Modal Decomposition (VMD) is an adaptive signal decomposition algorithm. It can decompose the signal into multiple components, and its essence and core idea is the construction and solution of the variational problem. VMD is commonly used to process non-linear signals and can decompose complex raw data to obtain a series of modal components^16^.

It can effectively extract the features of runoff data and reduce the influence of its nonlinearity and non-stationarity on the prediction results. The main steps of the VMD algorithm are: (1) The original signal is passed through the Hilbert transform to obtain a series of modal functions u, which are calculated to obtain the unilateral spectrum; (2) Transform the spectrum into the fundamental frequency band and construct the corresponding constrained variational problem by estimating the bandwidth; (3) Converting a constrained variational problem into an unconstrained variational problem^17^.

The calculated equations are as follows:

1 $$  L = \left( {\left\{ {u_{k} } \right\},\left\{ {\omega _{k} } \right\},\lambda } \right) = \alpha \mathop \sum \limits_{{k - 1}}^{K} \left\| {\partial _{t} \left[ {\left( {\delta \left( t \right) + \frac{j}{{\pi t}}} \right)*u_{k} \left( t \right)} \right]e^{{ - j\omega _{t} t}} } \right\|_{2}^{2}  + \left\| {f\left( t \right) - \mathop \sum \limits_{{k = 1}}^{K} u_{k} \left( t \right)} \right\|_{2}^{2}  + \left[ {\lambda \left( t \right),f\left( t \right) - \mathop \sum \limits_{{k = 1}}^{k} u_{k} \left( t \right)} \right],  $$

where ${u}_{k}\left(t\right)$ and ${\omega }_{k}$ are the modal components and the corresponding center frequencies, respectively, α is the penalty function and λ is the Lagrange multiplier. The results of several experiments show that the decomposition results are better when α is taken as 2000, so in this paper, α is set to 2000. The k modal components of the VMD are solved by using the alternating direction method of multiplicative operators to find the saddle points of the unconstrained variational problem.

There are some potential features of the VMD decomposed runoff residual sequence. The CEEMD decomposition method is a new adaptive signal processing method. Compared with the commonly used EEMD method, its decomposition efficiency and reconstruction accuracy are higher, and it better exploits the potential features of residual sequences.

The EMD method is a method proposed by Huang et al. for signal time-domain decomposition processing, which is particularly suitable for the analysis of nonlinear and non-stationary time series^18^. In order to cope with the modal confusion problem of the EMD method, Wu et al.^19^ proposed an overall average empirical modal decomposition. The EEMD method effectively suppresses the modal aliasing caused by the EMD method by adding white noise to the original signal several times, followed by EMD decomposition, and averaging the EMD decomposed IMFs as the final IMFs^20^.

CEEMD by adding two Gaussian white noise signals with opposite values to the original signal, which are then subjected to separate EMD decompositions. In ensuring that the decomposition effect is comparable to that of EEMD, CEEMD reduces the reconstruction error induced by the EEMD method. After the original signal x(t) is decomposed by CEEMD, the reconstructed signal can be represented as

2 $$x\left(t\right)=\sum_{i=1}^{n}IM{F}_{i}\left(t\right)+{r}_{n}\left(t\right)$$

In Eq. (2), $IM{F}_{i}\left(t\right)$ is the intrinsic modal function component; ${r}_{n}(t)$ is the residual term; and n is the number of intrinsic modal components when ${r}_{n}(t)$ becomes a monotonic function. The original sequence is finally decomposed into a finite number of IMFs.

### KELM

In order to accurately predict the runoff sequence, this paper establishes a kernel limit learning machine prediction model based on the kernel function optimised by the nature-inspired BOA algorithm.

**Figure 1:**
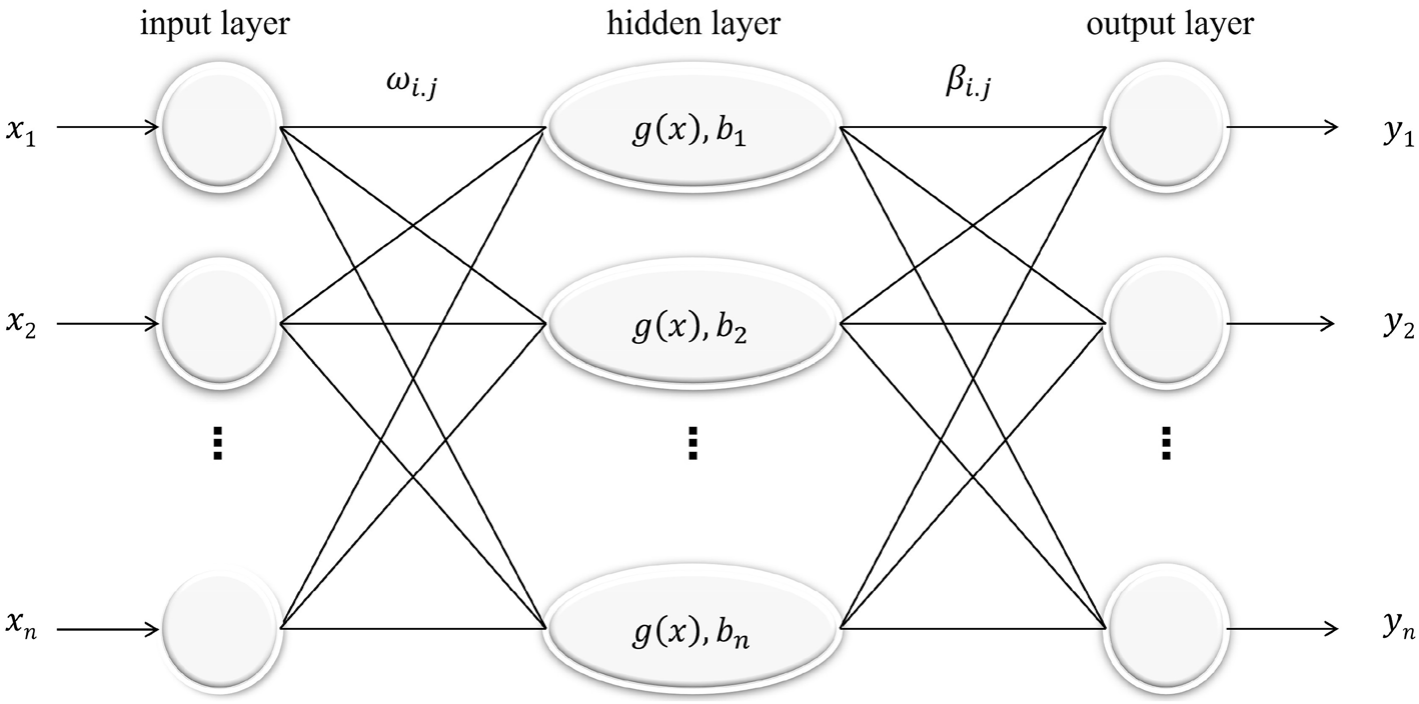
Structure of the KELM model.

In Fig. 1, the ELM input weights $\omega \in {R}^{XY}$ (X and Y are the input and hidden layer neural networks, respectively) and biases are randomly generated^21^. Extreme learning machines require less manual tuning of parameters than BP neural networks, and can be trained on sample data in a shorter period of time, with fast learning rate and strong generalisation ability.

Its regression function with output layer weights is:

3 $$\left\{\begin{array}{c}f\left(x\right)=h(x)\beta =H\beta \\ {{\varvec{H}}}^{T}{\left(\frac{1}{C}+{\varvec{H}}{{\varvec{H}}}^{T}\right)}^{-1}T\end{array}\right.$$

where: $f\left(x\right)$-model output; $x$ -sample input ${\varvec{h}}({\varvec{x}})$ and ${\varvec{H}}$-hidden layer mapping matrix; $\beta$ -regularisation parameter; T-sample output vector.

Conventional ELM prediction models (solved by least squares) tend to destabilise the output when there is potential covariance in the sample parameters. Therefore, Huang et al.^22^ used the Kernel Extreme Learning Machine (KELM) with kernel function optimisation. Based on the kernel function principle, KELM can project covariant input samples into a high-dimensional space, which significantly improves the fitting and generalisation ability of the model. In addition, this model does not need to set the number of hidden layer nodes manually, reducing the number of spatial training bits and training time. The model output equation is:

4 $$f\left(x\right)={\left[\begin{array}{c}K(x,{x}_{1})\\ \vdots \\ K(x,{x}_{N})\end{array}\right]}^{T}{\left(\frac{1}{C}+{{\varvec{\Omega}}}_{ELM}\right)}^{-1}$$

where: K(${x}_{i},{x}_{j}$)-kernel function; ${{\varvec{\Omega}}}_{ELM}$-kernel matrix, which is calculated as:

5 $$\left\{\begin{array}{c}{{\varvec{\Omega}}}_{ELM}=H{{\varvec{H}}}^{T}\\ {{{\varvec{\Omega}}}_{ELM}}_{i,j}=h\left({x}_{i}\right)h\left({x}_{j}\right)=K\left({x}_{i},{x}_{j}\right)\end{array}\right.$$

where: ${x}_{i}$ and ${x}_{j}$-sample input vectors, i and j are taken as positive integers within [1,N]; K(${x}_{i},{x}_{j}$)-kernel function.

KELM determines the implicit layer mapping kernel function in the form of an inner product by introducing a kernel function, and the number of implicit layer nodes does not need to be set; The result is faster model learning and effective improvement of the generalisation ability and stability of the KELM-based runoff prediction model.

### BOA optimisation of KELM

Butterfly optimisation algorithm is an intelligent optimisation algorithm derived by simulating butterfly searching for food and mating behaviour^23^. In the BOA algorithm, each butterfly emits its own unique scent. Butterflies are able to sense the source of food in the air and likewise sense the scent emitted by other butterflies and move with the butterfly that emits a stronger scent, the scent concentration equation is:

6 $$f=c{l}^{a}$$

where $f$—Concentration of scent emitted by the butterfly, $c$—Perceived morphology, $l$—Stimulus intensity, $a$—Power index, taken between [0,1]. When a = 1, it means that the butterfly does not absorb the scent, meaning that the scent emitted by a specific butterfly is perceived by the same butterfly; This case is equivalent to a scent spreading in an ideal environment, where the butterfly emitting the scent can be sensed everywhere in the domain, and thus a single global optimum can be easily reached.

In order to prove the above with the search algorithm, the following hypothetical regulations were set up to idealise the characteristics of butterflies: (i) All butterflies can give off some scent, and butterflies attract and exchange information with each other by virtue of the scent. (ii) Butterflies undergo random movements or directional movements towards butterflies with strong scent concentrations.

By defining different fitness functions for different problems, the BOA algorithm can be divided into the following 3 steps:

Step 1: Initialisation phase. Randomly generate butterfly locations in the search space, calculate and store each butterfly location and fitness value.

Step 2: Iteration phase. Multiple iterations are performed by the algorithm, in each iteration the butterflies are moved to a new position in the search space and then their fitness values are recalculated. The adaptation values of the randomly generated butterfly population are sorted to find the best position of the butterfly in the search space.

Step 3: End Phase, In the previous phase, the butterflies move and then use the scent formula to produce a scent in a new location.

The penalty parameter C and the kernel function parameter K in the kernel-limit learning machine are chosen as the searching individuals of the butterfly population, and the BOA-KELM model is constructed to achieve the iterative optimisation of C and K. The specific steps are as follows:

Step 1: Collect runoff data and produce training and prediction sample sets.

Step 2: Initialise the butterfly population searching individuals i.e. penalty parameter C and kernel function parameter K.

Step 3: Initialise the algorithm parameters, including the number of butterfly populations M, the maximum number of iterations .

Step 4: Calculate the fitness value of the individual butterfly population and calculate the scent concentration f. Based on the fitness value, the optimal butterfly location is derived.

Step 5: Check the fitness value of the butterfly population searching individuals after updating their positions, determine whether it is better than before updating, and update the global optimal butterfly position and fitness value.

Step 6:Judge whether the termination condition is satisfied. If it is satisfied, exit the loop and output the prediction result; otherwise, bring in the calculation again.

Step 7:Input the test set into the optimised KELM and output the predictions.

**Figure 2:**
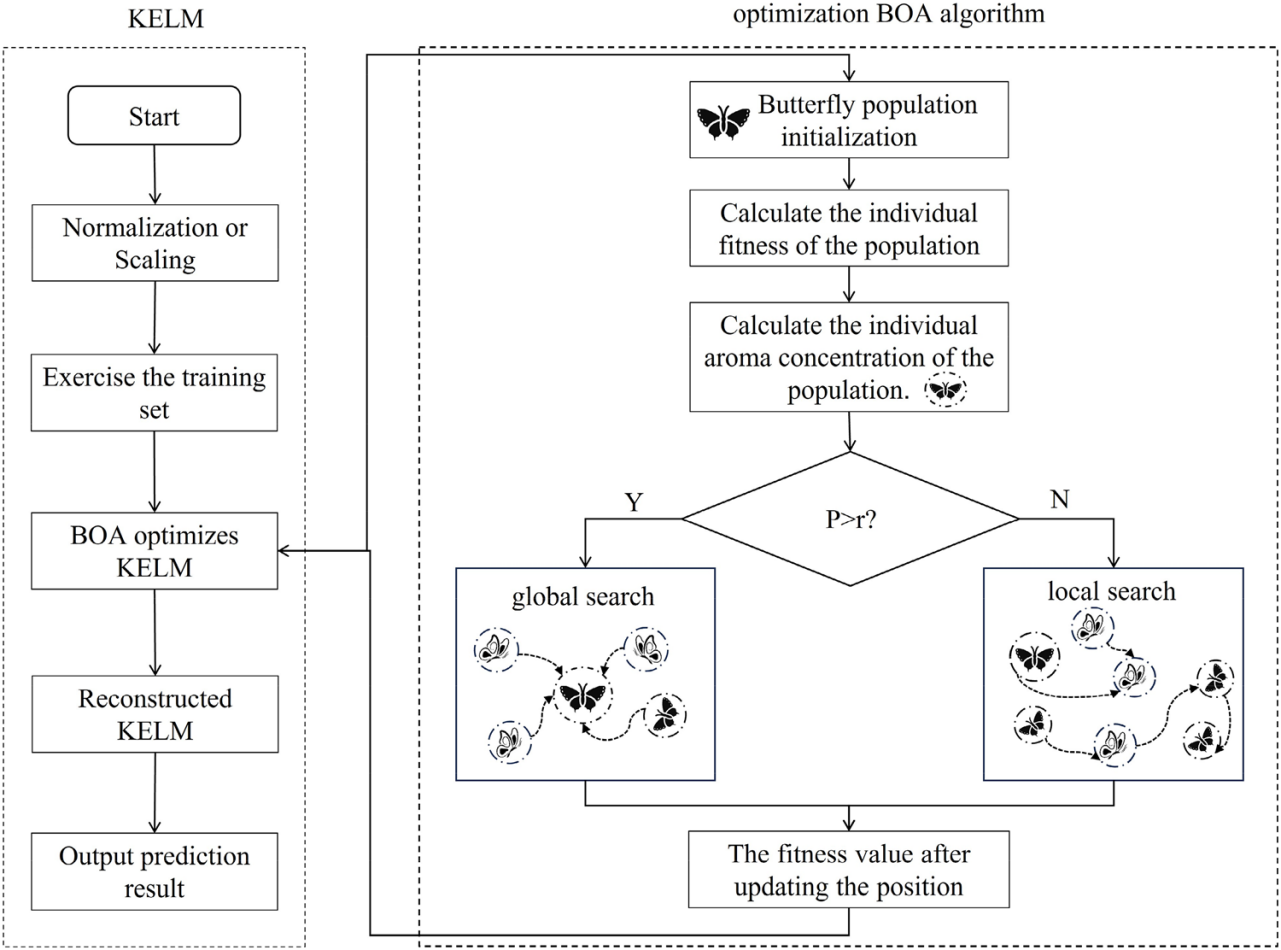
BOA Optimisation KELM Model Flowchart.

According to the above steps, the corresponding flowchart is shown in Fig. 2.

### VMD-CEEMD-BOA-KELM prediction model

**Figure 3:**
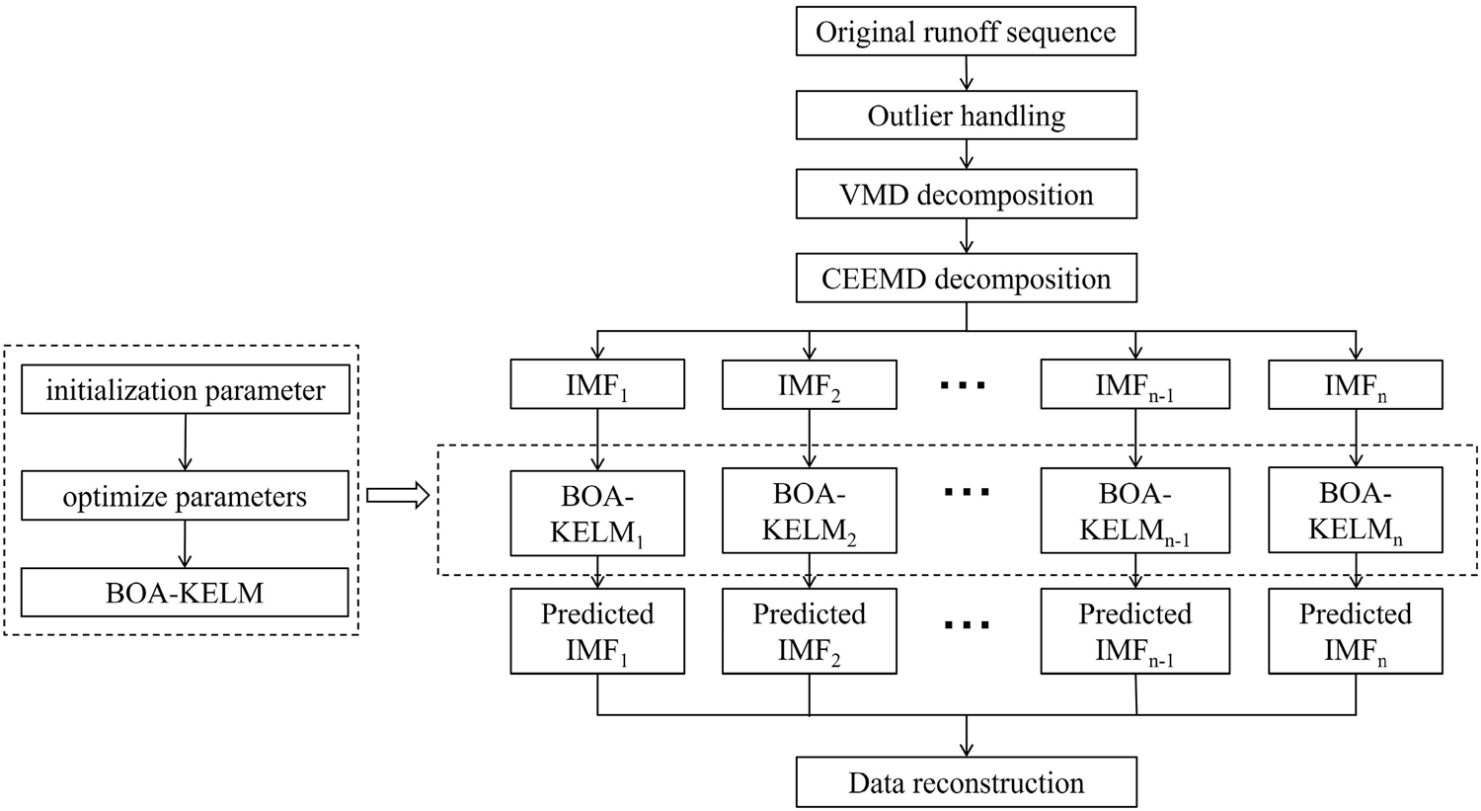
VMD-CEEMD-BOA-KELM prediction model framework.

In order to improve the accuracy of runoff prediction, this paper designs a runoff prediction framework based on the idea of "decomposition—modeling prediction—reconstruction", as shown in Fig. 3, and the specific prediction steps are as follows:

Step 1: Data pre-processing. Anomalies in the original runoff series were processed using the Lajda criterion.

Step 2: VMD-CEEMD decomposition. The raw runoff series was decomposed using the VMD algorithm, and then the data was decomposed quadratically using the CEEMD algorithm to obtain k components.

Step 3: Data preparation. Each component is normalised and divided into a training data set and a test data set.

Step 4: Modelling prediction. A BOA-optimised KELM model is built based on the training dataset for each component and predicted for the test dataset.

Step 5: Reconstruction. The predictions of all components are accumulated to obtain the prediction of the original runoff sequence.

### Evaluation indicators

In order to reflect the error and prediction accuracy of the model prediction results more clearly, four indicators, RMSE, MAE, R^2^, and NSE are used for the analysis, and the equations are calculated as follows:

$${\varvec{R}}{\varvec{M}}{\varvec{S}}{\varvec{E}}=\sqrt{\frac{1}{{\varvec{N}}}\cdot {\sum }_{{\varvec{i}}=1}^{{\varvec{N}}}{\left({{\varvec{y}}}_{{\varvec{i}}}-{{\varvec{y}}}_{{\varvec{c}}}\right)}^{2}}$$

$${\varvec{M}}{\varvec{A}}{\varvec{E}}=\frac{1}{{\varvec{N}}}\cdot {\sum }_{{\varvec{i}}=1}^{{\varvec{N}}}\left|{{\varvec{y}}}_{{\varvec{i}}}-{{\varvec{y}}}_{{\varvec{c}}}\right|$$

$${{\varvec{R}}}^{2}={\left[\frac{\sum \left({{\varvec{y}}}_{{\varvec{i}}}-\overline{{{\varvec{y}} }_{{\varvec{i}}}}\right)\left({{\varvec{y}}}_{{\varvec{c}}}-\overline{{{\varvec{y}} }_{{\varvec{c}}}}\right)}{\sqrt{\sum {\left({{\varvec{y}}}_{{\varvec{i}}}-\overline{{{\varvec{y}} }_{{\varvec{i}}}}\right)}^{2}}\sum {\left({{\varvec{y}}}_{{\varvec{c}}}-\overline{{{\varvec{y}} }_{{\varvec{c}}}}\right)}^{2}}\right]}^{2}$$

$${\varvec{N}}{\varvec{S}}{\varvec{E}}=1-\frac{{\sum }_{{\varvec{t}}=1}^{{\varvec{T}}}{\left({{\varvec{y}}}_{{\varvec{i}}}-{{\varvec{y}}}_{{\varvec{c}}}\right)}^{2}}{{\sum }_{{\varvec{t}}=1}^{{\varvec{T}}}{\left({{\varvec{y}}}_{{\varvec{i}}}-\overline{{{\varvec{y}} }_{{\varvec{i}}}}\right)}^{2}}$$

### Ethical approval

This paper does not contain any studies with human participants or animals performed by any of the authors.

## Example applications

### Data sources

**Figure 4:**
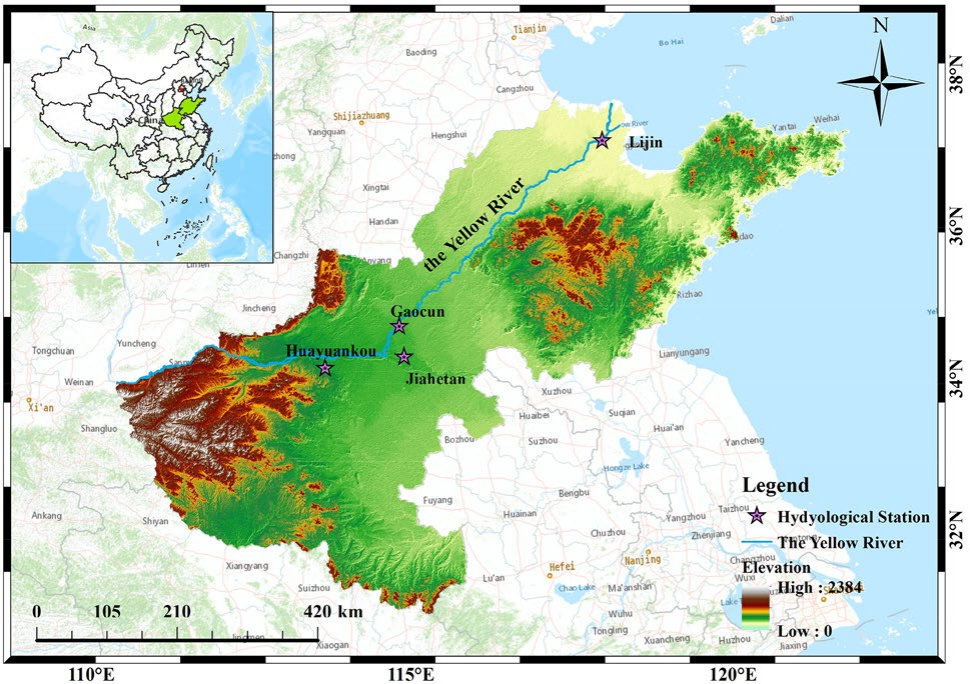
Location map of the study area.

The study area of this paper is Jiehetan, Huayuankou, Gaocun and Lijin hydrological stations, and the data are all obtained from the measured data of the hydrological stations in the Yellow River Basin and have been checked for tricity. The location of the study area is shown in Fig. 4. This map was created using the ArcMap 10.2 URL: www.arcgis.com.

**Figure 5:**
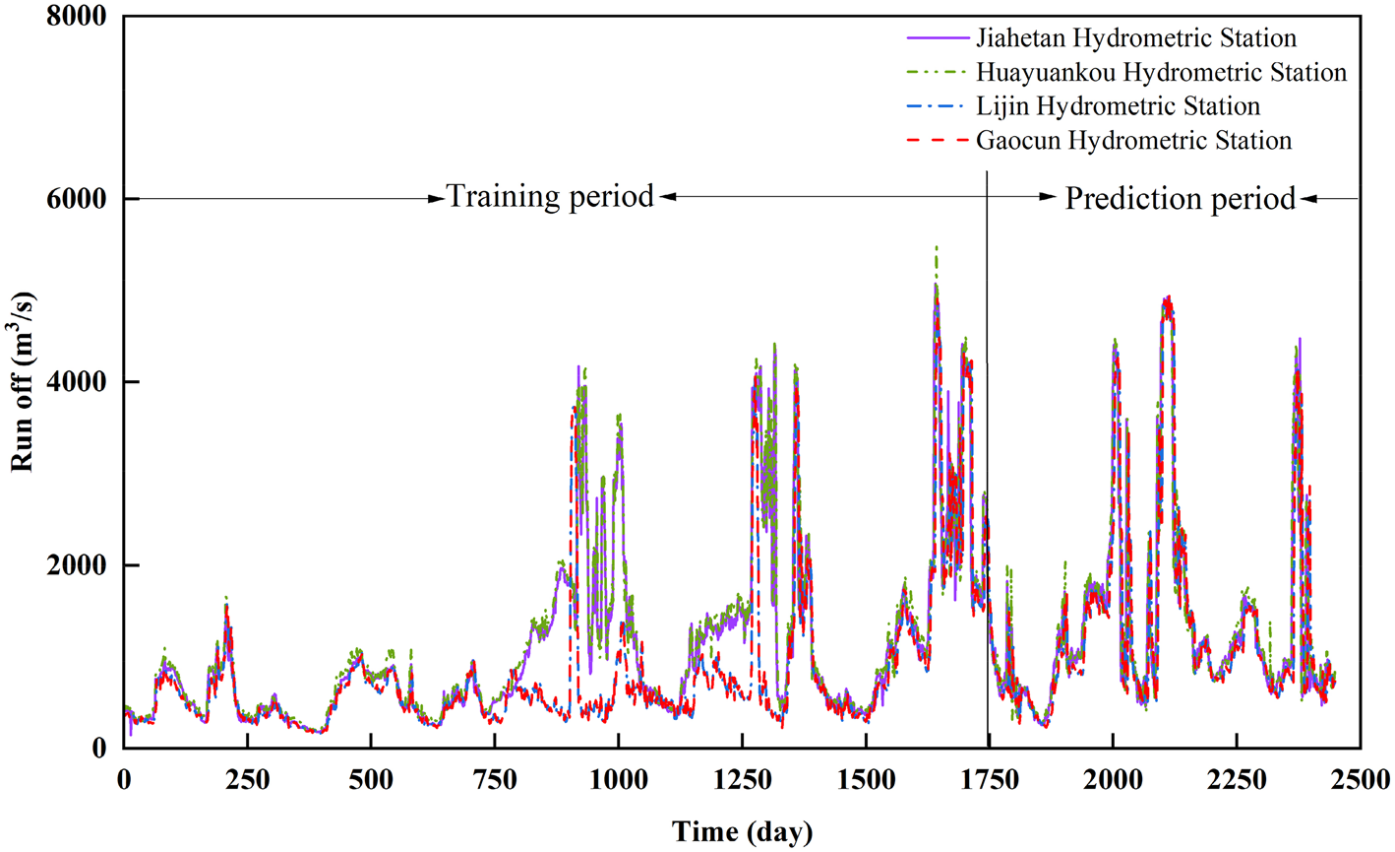
Daily runoff series graph.

The day-by-day runoff sequences from four hydrological stations in the Yellow River Basin for the period of 2016–2022 were selected for the experiments, and the first 70% of the data were classified as the training sample set, and the remaining 30% of the data were classified as the test set, and the process of the daily runoff sequences is shown in Fig. 5.

### Data decomposition

**Figure 6:**
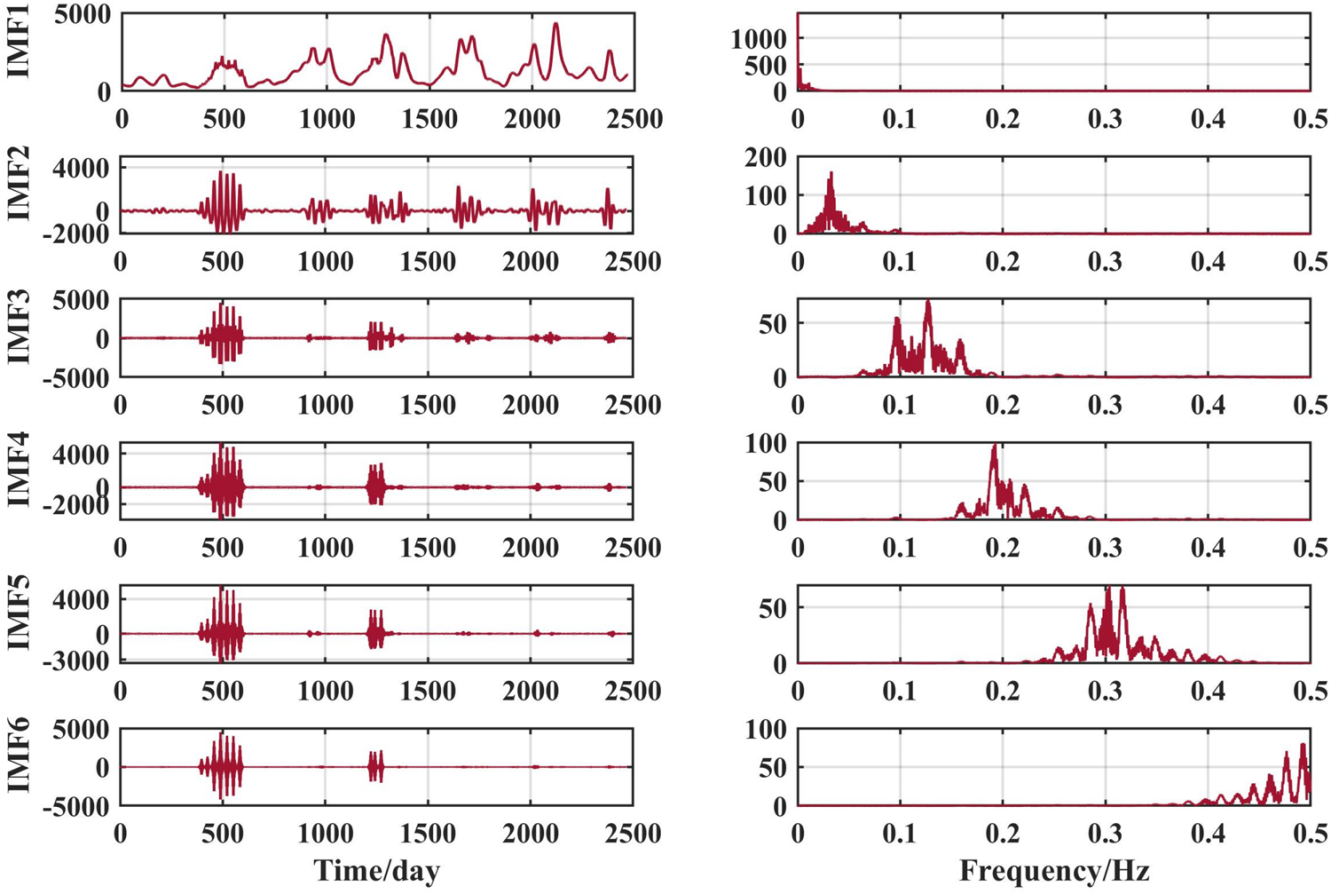
VMD decomposition results for Jiahetan hydrological station.

**Figure 7:**
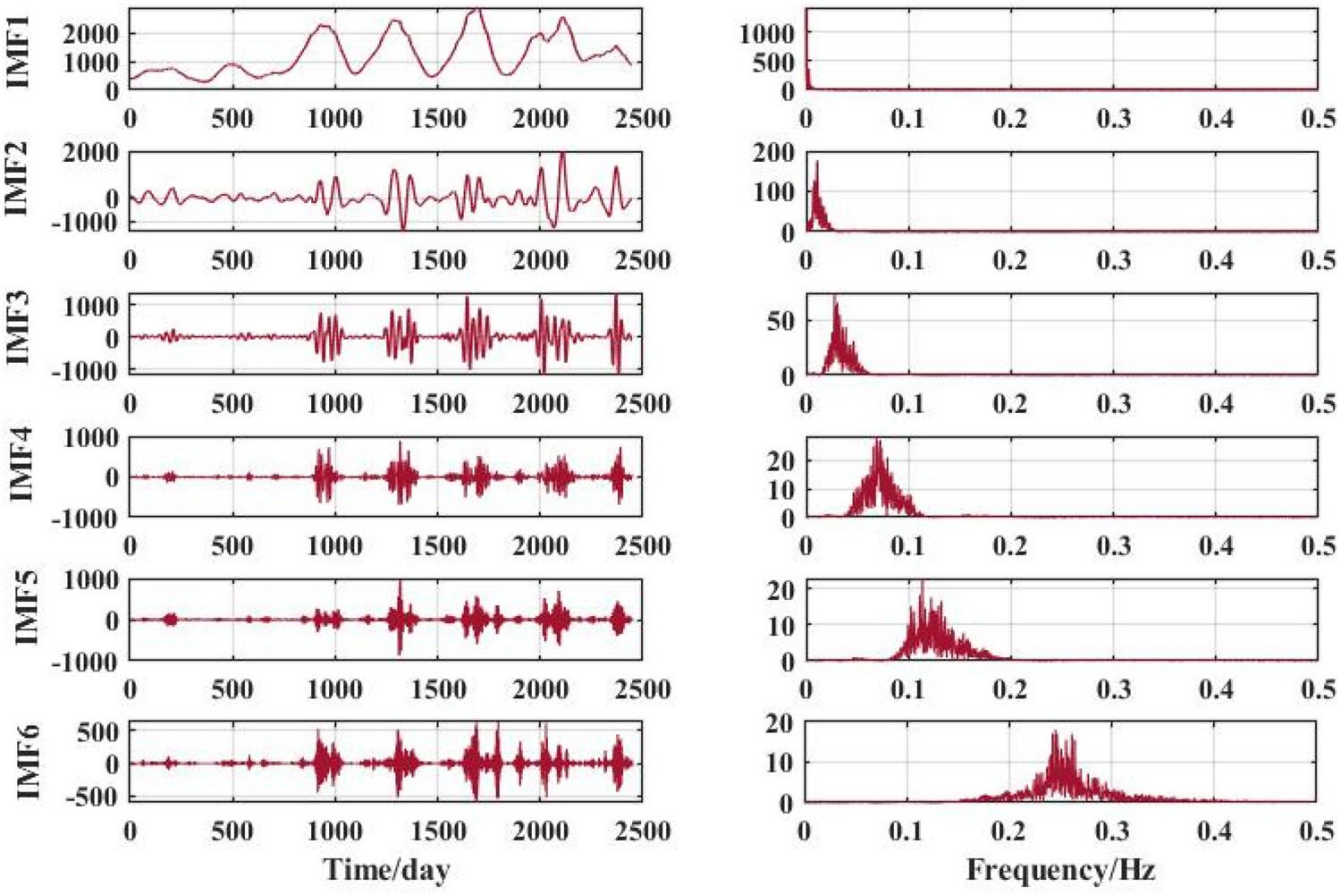
VMD decomposition results for Huayuankou hydrological station.

**Figure 8:**
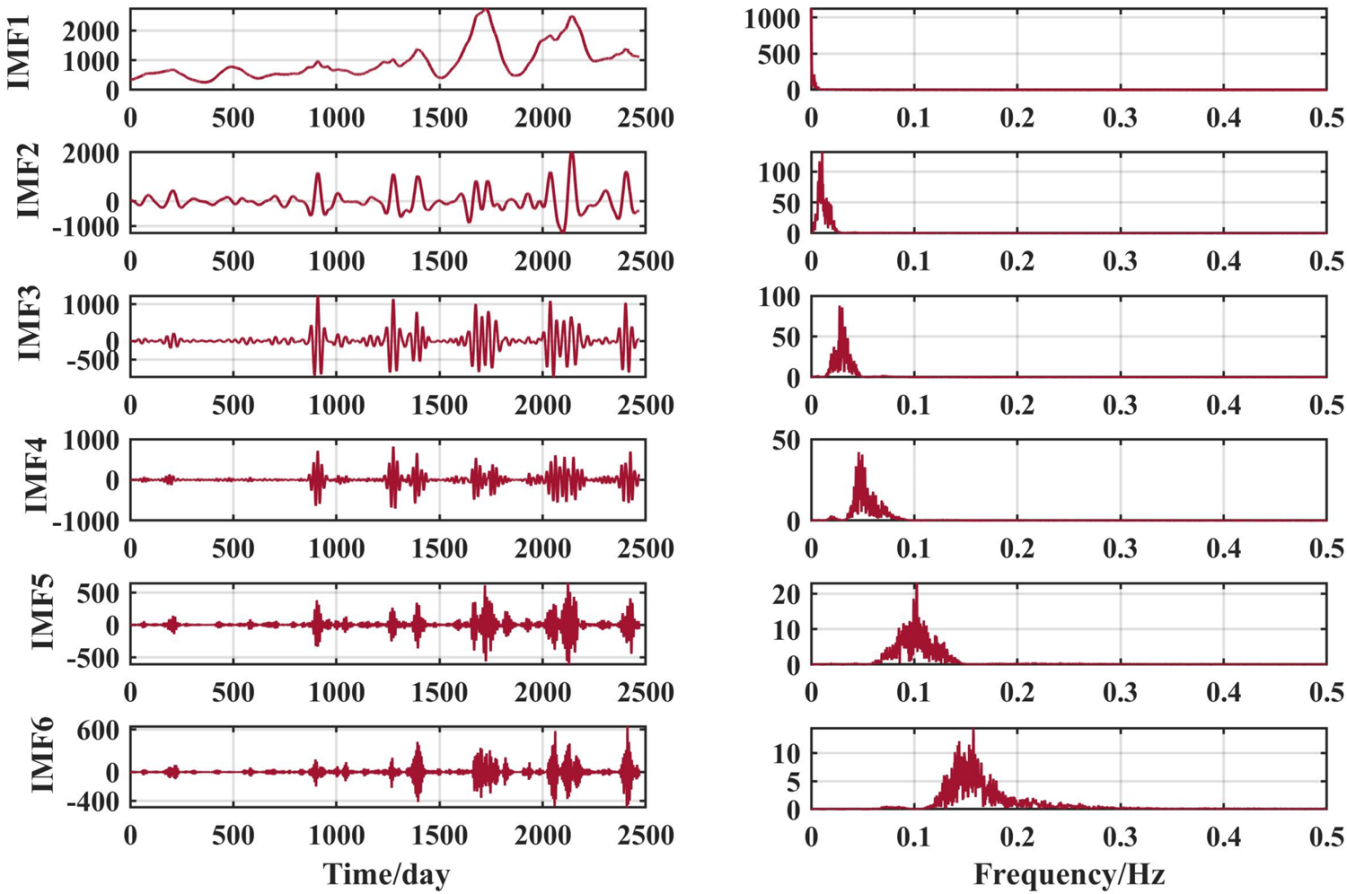
VMD decomposition results for Gaocun hydrological station.

**Figure 9:**
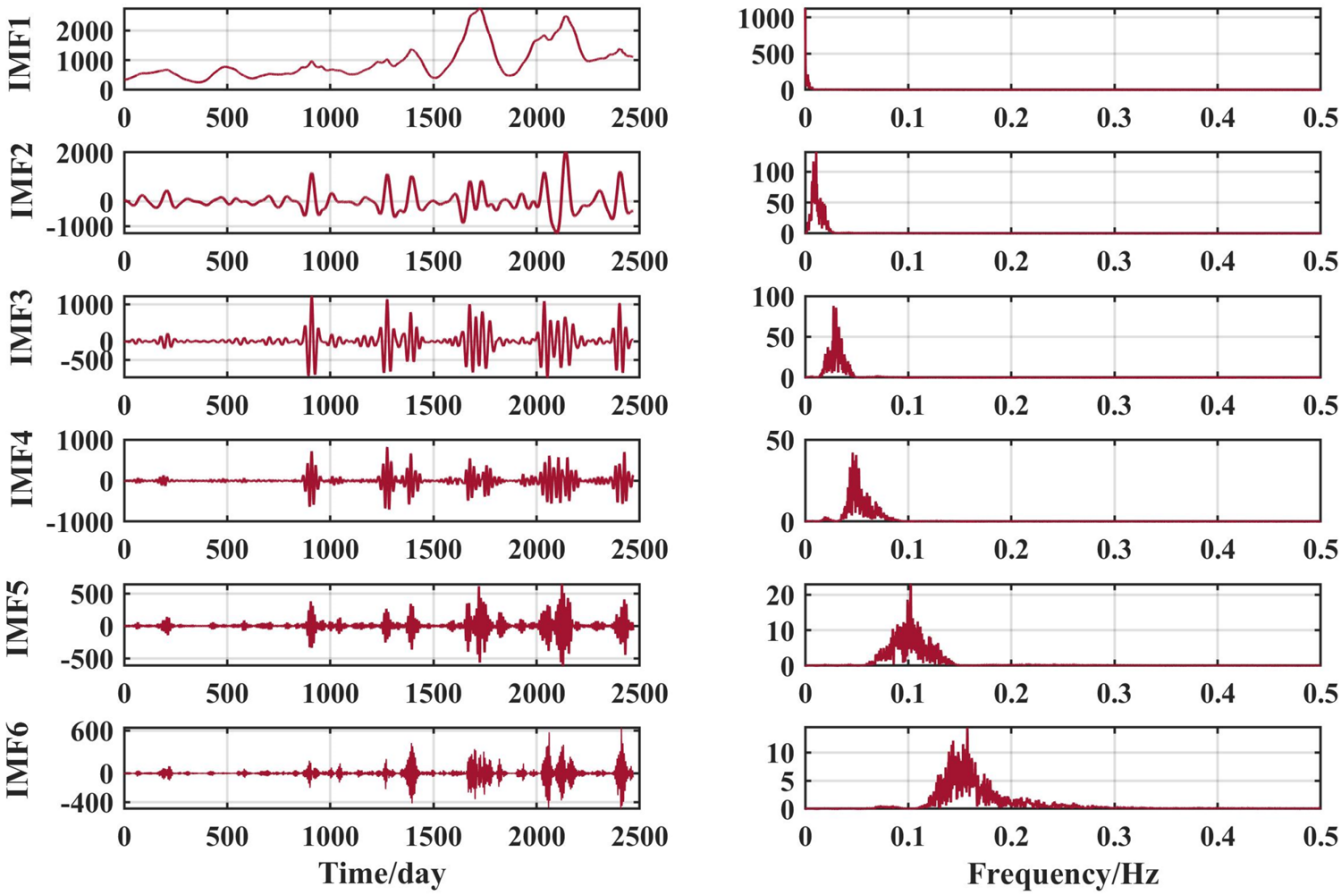
VMD decomposition results for Lijin hydrological station.

The above runoff sequence was decomposed using the VMD algorithm to obtain six components IMF1 to IMF6, as shown in Figs. 6, 7, 8 and 9.

The VMD decomposition method is used to decompose the raw runoff series to visualise the hidden information such as the cyclical trend inherent in the time series, and at the same time increase the amount of data information for the prediction model. Long-term trend changes, periodic changes and irregular random change sequences were obtained. The fluctuations of the residual terms decomposed from the runoff series showed randomness and the fluctuations increased significantly with the onset of the flood season each year.

The choice of the number of different modes k affects the results of the VMD decomposition and also the final prediction. If the number of components of the decomposition is too small, the accuracy of the decomposition is not guaranteed and cannot effectively reduce the complexity of the original sequence; If the number of components is high, it results in some of the modes having the same frequency and produces an over-decomposition. In this paper, the optimal k value is obtained adaptively after permutation entropy algorithm to obtain six sequence components.

**Figure 10:**
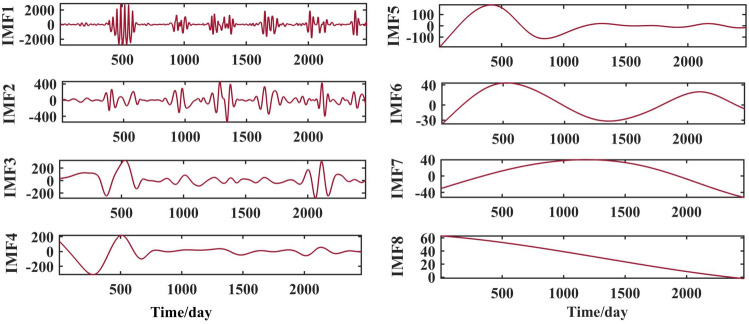
CEEMD decomposition results for Jiahetan hydrological station.

**Figure 11:**
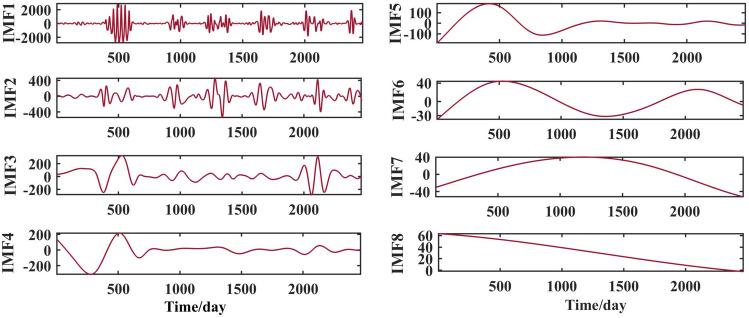
CEEMD decomposition results for Huayuankou hydrological station.

**Figure 12:**
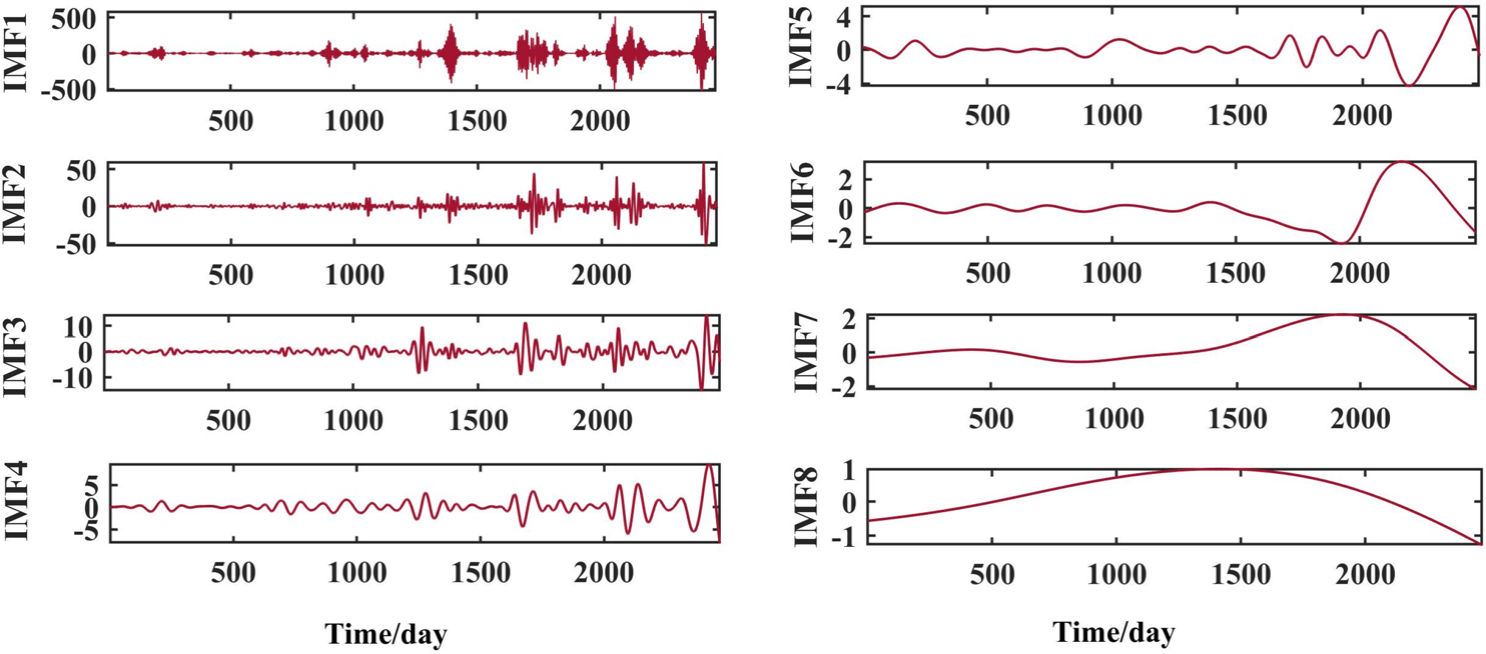
CEEMD decomposition results for Gaocun hydrological station.

**Figure 13:**
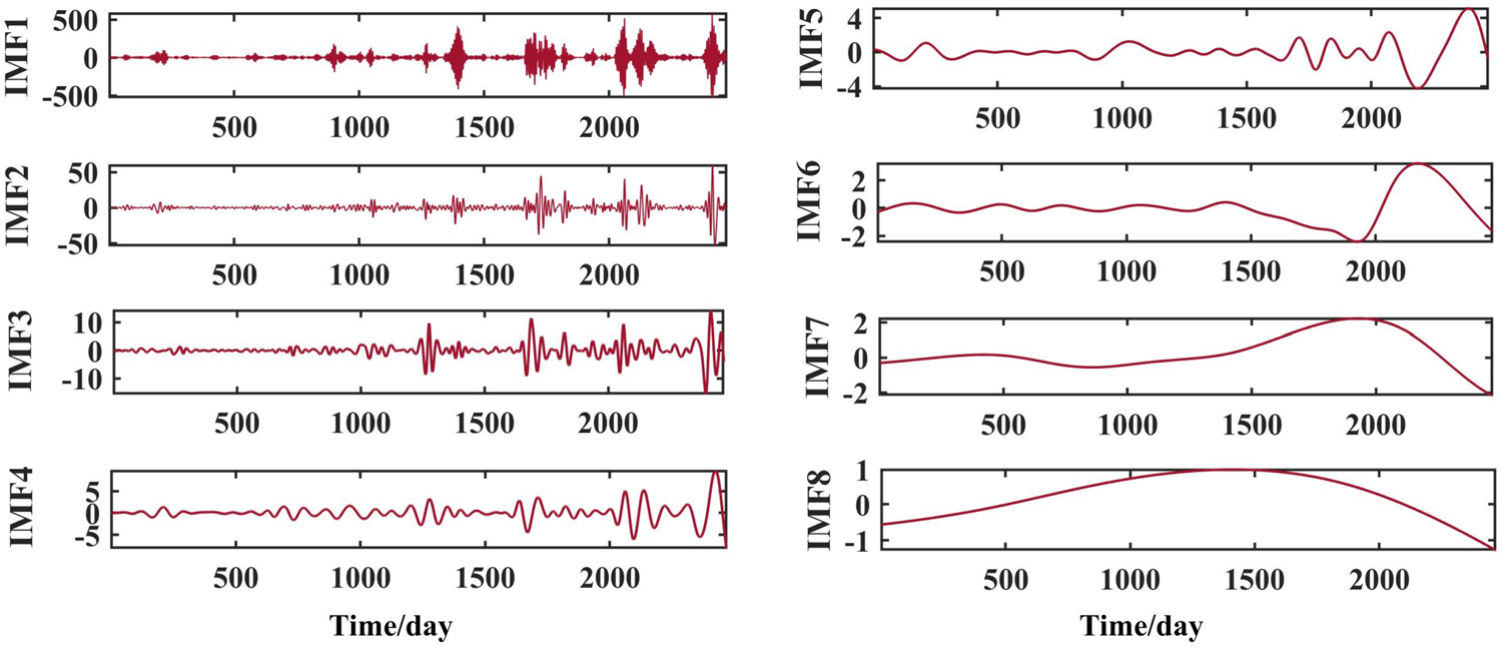
CEEMD decomposition results for Lijin hydrological station.

After selecting the number of modes, the raw runoff sequence was decomposed by VMD into six decomposition results with high complexity. From the above Fig. 6, it can be seen that the IMF2 term after the VMD decomposition of the Clipper Beach station has a strong volatility, and its sample entropy^24^ value is calculated to be 1.5649. From Figs. 7, 8, and 9, it can be seen that the IMF8 terms after VMD decomposition of Huayuankou, Gaocun, and Lijin stations have strong volatility, are more complex, and carry rich information. If they are predicted directly during the modeling process, the predictive accuracy of the overall model will be weakened. The EEMD quadratic decomposition is performed on these highly volatile terms, and the results of the decomposition are shown in Figs. 10, 11, 12, and 13.

When analyzed together with the above decomposition diagrams, both decomposition methods can effectively separate the frequency, amplitude and period contained in the runoff and reduce the non-linear characteristics of the runoff. In the VMD decomposition results, some signals with similar scales are present in some epochs of IMF4 and IMF5, suggesting that modal mixing may occur in these three components; In the CEEMD decomposition results, there are no signals with very different eigentime scales in the same IMF component, and there are no signals with similar scales in different IMF components, indicating that the decomposition method avoids the phenomenon of modal aliasing.

### Inputs and outputs of the predictive model

For each of the above IMFs, a BOA-KELM prediction model is built separately, and the superposition of the prediction results of each sub-sequence is the prediction result of the original runoff sequence. Where the input step of the model is determined using a partial auto correlation function (PACF) that highlights the effect of time lag on runoff in the current time period. Assuming the output variable is ${x}_{i}$, the first L variables are the input variables when the PACF of lag L exceeds the 95% confidence interval ^25^.

**Figure 14:**
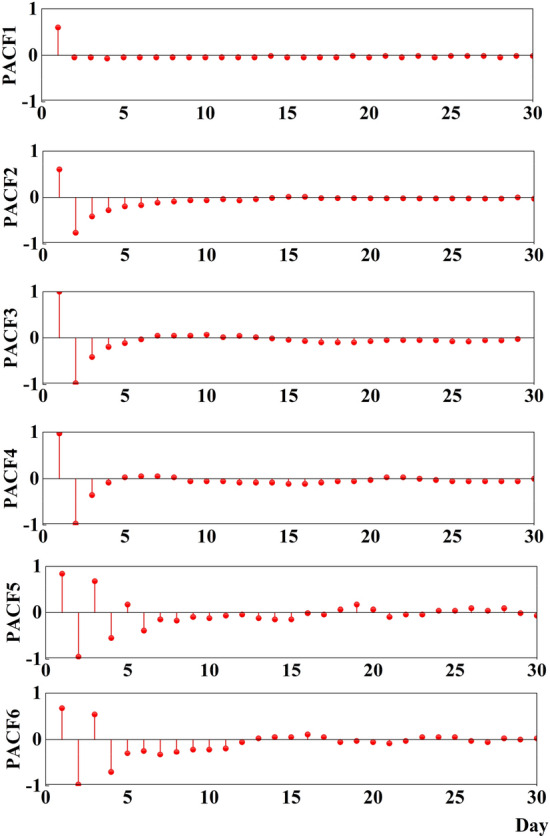
PACF for runoff sequences.

Taking the Clip River Beach station as an example, the original daily runoff sequence was decomposed by VMD to obtain six sub-sequences, and the input steps of each IMF were calculated by PACF as 1, 6, 4, 4, 8, and 11, respectively. The Clipper Beach runoff sequence PACF is shown in Fig. 14, and the specific input step and input variables for each hydrological station are shown in Table 1.

**Table 1:** Input dimensions of the prediction model for each component.

Component		IMF1	IMF2	IMF3	IMF4	IMF5	IMF6
Dimension of input	Jiahetan Hydrometric Station	1	6	4	4	8	11
	Huayuankou Hydrometric Station	1	1	3	4	8	8
	Gaocun Hydrometric Station	1	2	4	6	8	10
	Lijin Hydrometric Station	1	1	3	4	6	9

As can be seen in Fig. 14, among all these delays, one of the delays in PACF1 exceeds the threshold corresponding to the 95% confidence interval, and therefore the input dimension of the prediction model for direct prediction of runoff sequences is taken to be 1.

## Discussion

### Analysis of the results of the VMD-CEEMD-BOA-KELM prediction model

**Figure 15:**
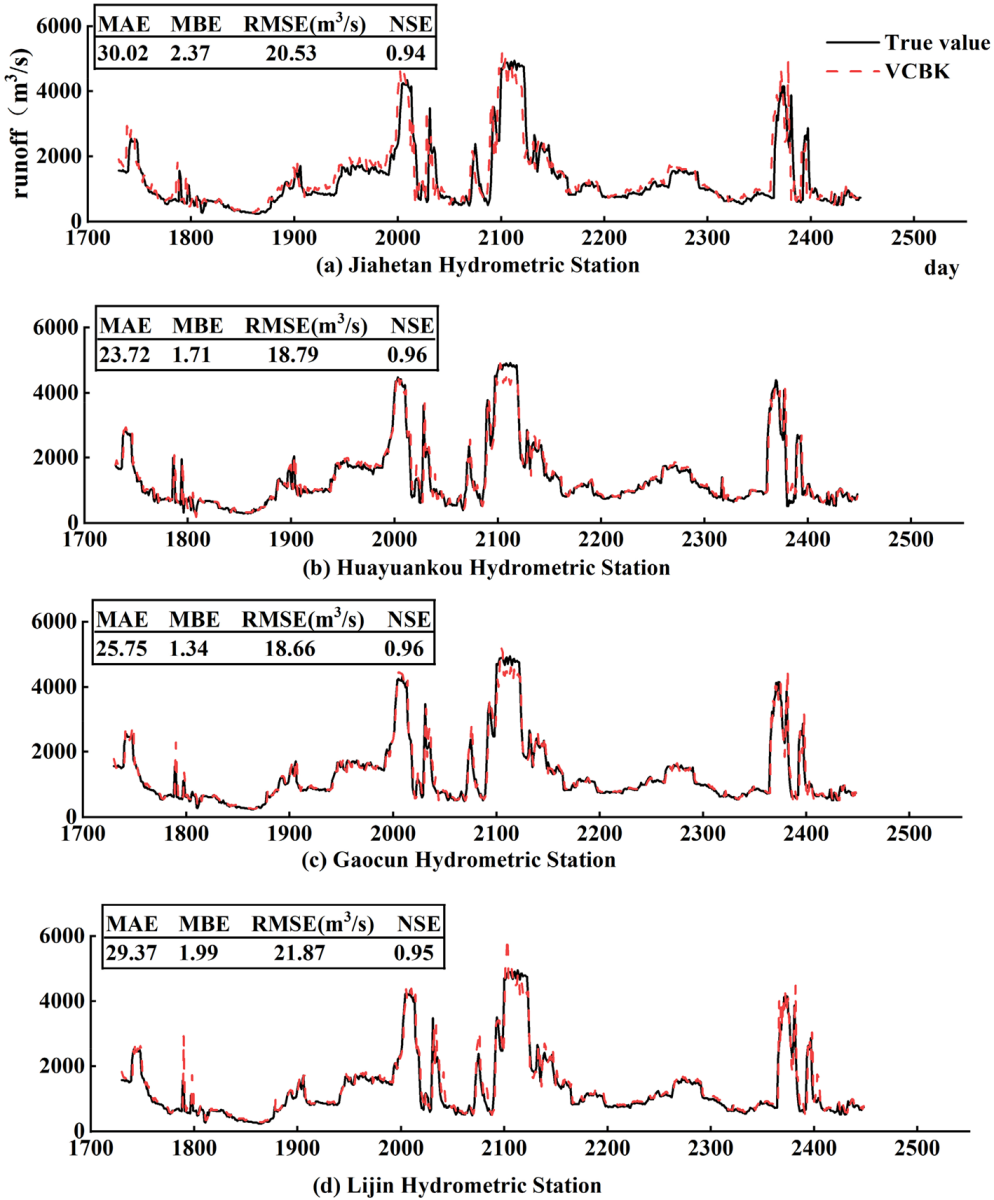
VCBK model prediction results.

Combining the multi-step decomposition and butterfly optimisation algorithms to improve the kernel limit learning machine to obtain the four hydrological stations runoff sequence prediction results are shown in Fig. 15. It can be seen that the VMD-CEEMD-BOA-KELM (VCBK) model achieves a better fit. The NSE values of their test sets when using the kernel function are all higher than 0.9, the MAEs of the four hydrological stations were 30.02, 23.72, 25.75, and 29.37, the MBEs were 2.37, 1.71, 1.34, and 1.99, and the RMSEs were 20.53 m^3^ /s, 18.79 m^3^ /s, 18.66 m^3^ /s, and 21.87 m^3^ /s, respectively. The predicted values of the VCBK model are closer to the true values of the samples and have high accuracy.

### Comparative analysis with other models

**Figure 16:**
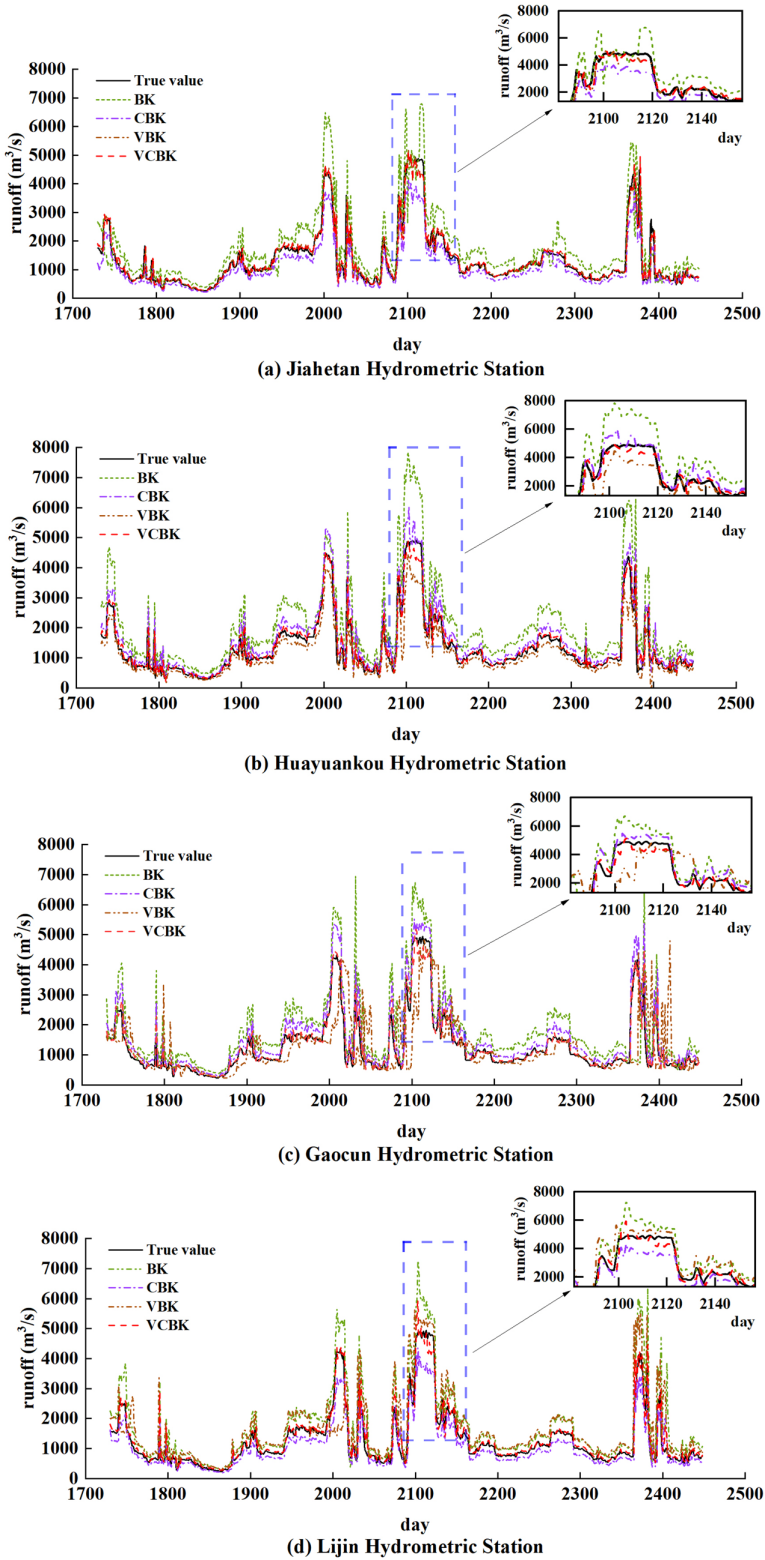
Model predictions for each hydrological station.

In this paper, BOA-KELM (BK) without decomposition of the real sequence, VMD- BOA-KELM (VBK) after VMD decomposition, and CEEMD-BOA-KELM (CBK) after CEEMD decomposition are selected as the comparative models, and the prediction results of each model are shown in Fig. 16.

As can be seen from Fig. 16, the prediction curves of the four models at the rest of the time are overall similar to the trend of the measured value curves, except for the time period corresponding to the rectangular area. At all time points, the predicted values of the VCBK model are infinitely close to the measured values, and therefore it has the highest prediction accuracy, while the CBK and VBK models have higher prediction accuracy, and the BK model has the lowest prediction accuracy. Further, zooming in on the prediction curves in the rectangular region, as shown in the small graph in Fig. 16, it can be seen that the prediction curves of the VCBK model, the VBK model, and the CBK model combined with the data decomposition algorithm are closer to the measured values than the prediction curves of the BK model without the data decomposition algorithm combined, it shows that the hybrid runoff prediction model combining decomposition methods can improve the prediction accuracy of the peak point of the runoff sequence. The error metrics MAE, MBE, RMSE and NSE of the statistical four prediction models are shown in Table 2.

**Table 2:** Table of prediction errors.

Hydrometric Station	Model	MAE(m^3^/s)	R^2^	RMSE(m^3^/s)	NSE
Jiahetan Hydrometric Station	BK	86.6	0.79	86.55	0.73
	VBK	63.21	0.88	50.67	0.86
	CBK	45.65	0.91	34.12	0.9
	VCBK	30.02	0.94	20.53	0.94
Huayuankou Hydrometric Station	BK	104.84	0.70	94.56	0.69
	VBK	58.28	0.86	68.22	0.84
	CBK	47.18	0.88	35.68	0.87
	VCBK	23.72	0.96	18.79	0.96
Gaocun Hydrometric Station	BK	84.16	0.80	84.56	0.71
	VBK	56.17	0.85	48.79	0.82
	CBK	34.51	0.93	28.47	0.91
	VCBK	25.75	0.98	18.66	0.96
Lijin Hydrometric Station	BK	107.51	0.75	100.65	0.67
	VBK	49.48	0.9	60.32	0.87
	CBK	34.48	0.93	39.88	0.92
	VCBK	29.37	0.97	21.87	0.95

**Figure 17:**
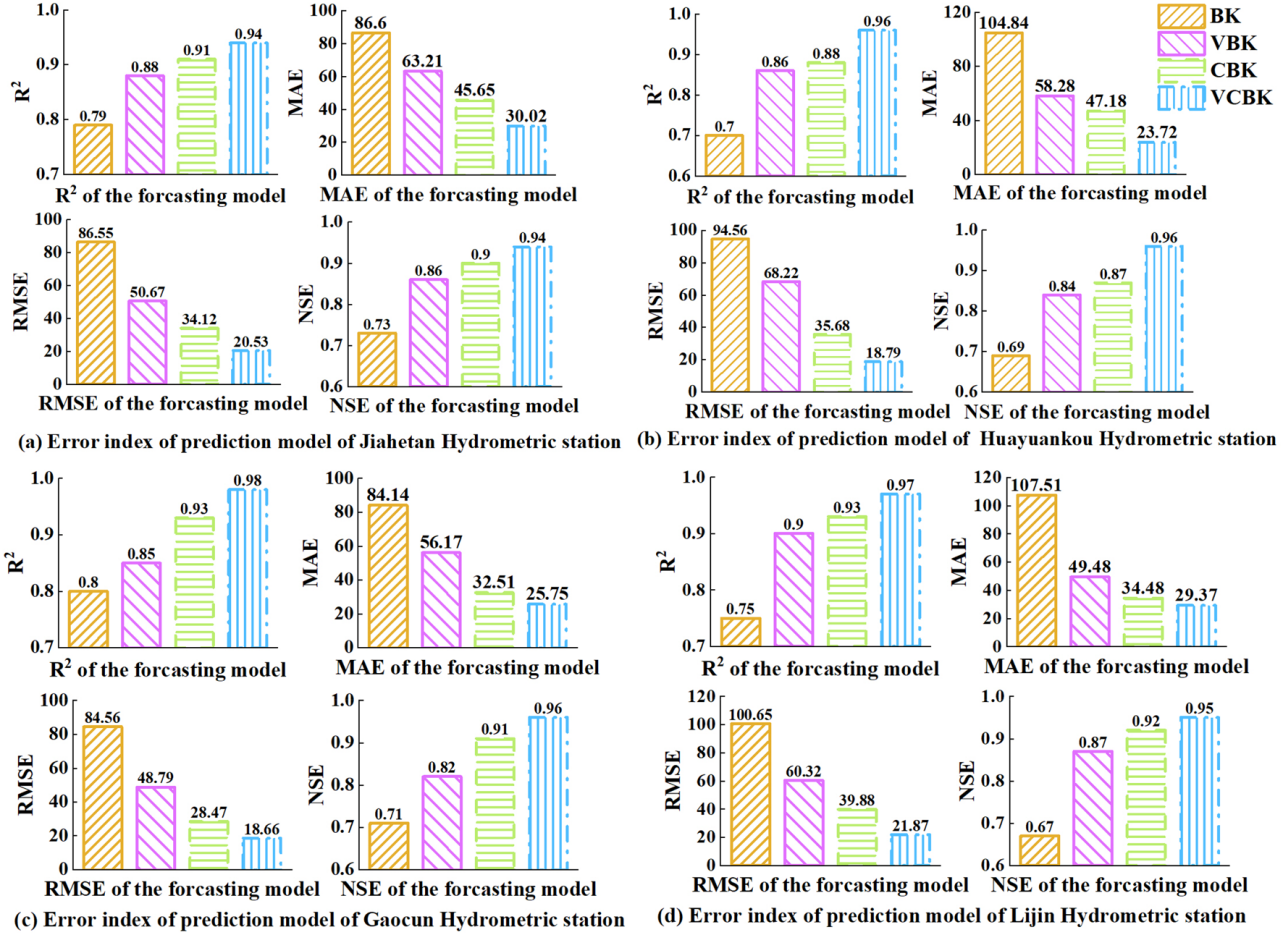
Indicators of modeling error for each hydrological station.

From the results in Table 2, it can be found that the prediction performance of the VCBK model after quadratic decomposition is optimal, and the NSE, RMSE, MAE, and R^2^ evaluation indexes are improved compared with the other comparison models. The values of the four error indicators of the BK model are inferior to those of the VBK and CBK models, indicating that the prediction accuracy of the decomposed model is better than that of the undecomposed model. For example, the MAE of the BK model without the combined data decomposition algorithm is 86.6, R^2^ is 0.79, RMSE is 86.55 m^3^/s, and NSE is 0.73 for the Jiehetan station. The MAE of both models combined with the data decomposition algorithm was lower than 80, the R^2^ was higher than 0.80, the RMSE was lower than 60 m^3^/s, and the NSE was higher than 0.8. Further comparison of the values of the error metrics of the VCBK model with those of the VBK model and the CBK model shows that the values of the four error metrics of the VCBK model are superior to those of the VBK model and the CBK model. It shows that the prediction model after secondary decomposition has better prediction performance than the model with only one decomposition. In order to have a more intuitive understanding of the prediction effect of the models, the histograms of the four error indicators of the four prediction models are shown in Fig. 17.

Taking the Clipper Beach station as an example, it can be seen from Fig. 17a that (1) the VCBK hybrid model reduces MAE by 65.33%, R^2^ increased by 15 per cent, RMSE by 76.27%, and NSE improves by 22.34% compared with the BK model without sequence decomposition. (2) The VCBK hybrid model reduces MAE by 52.50 per cent, R^2^ increased by 6 per cent, RMSE by 59.48 per cent and NSE by 8.51 per cent compared to the VBK model. (3) The VCBK hybrid model reduces MAE by 34.24 per cent, R^2^ increased by 3 per cent, RMSE by 39.83 per cent and NSE by 4.25 per cent compared to the CBK model.

From the above, it can be seen that the combined model is much better than the single model, both in terms of forecasting accuracy and prediction error. The combined model VCBK model has the best goodness of fit, and the quadratic decomposition for preprocessing is better in the prediction process of these four sites when comparing the combined model VBK and the CBK model. The variational modal decomposition decomposition method is a completely non-recursive decomposition method, which can effectively reduce the complexity of the runoff sequence. The CEEMD algorithm is used to reduce the instability of the runoff sequence by further decomposing the random component with the largest frequency into a number of components with different frequencies that are more stable than the random component. The butterfly optimisation algorithm is also used to find the globally optimal parameters so that the kernel-limit learning machine is able to provide a better prediction of the smoothed runoff. In summary, the combination of variational modal decomposition and complementary ensemble empirical modal decomposition with the kernel-limit learning machine model of the butterfly optimisation algorithm can effectively predict runoff sequences of high complexity.

## Conclusion

1. In the prediction of daily runoff at the four hydrological stations, the single forecast model showed a large difference between the true and predicted values at some points, which led to a high prediction error. This is due to the non-stationary, non-linear nature of the runoff, so it is necessary to pre-process the runoff and perform a multi-step decomposition of the runoff sequence. Compared with the individual network models, the prediction effect is significantly improved, and the prediction accuracy of the model with quadratic decomposition is significantly improved at the peaks of the runoff series compared with the model without quadratic decomposition.
2. The VCBK combined forecast model was applied to the daily runoff forecasts at four hydrological stations in the Yellow River Basin and compared with other combined forecast models, and the values of the four error indexes of the VCBK model were better than those of the BK model, the VBK model and the CBK model.
3. The model proposed in this paper, which combines the variational modal decomposition and the complementary ensemble empirical modal decomposition with the kernel-limit learning machine model of the butterfly optimisation algorithm, can effectively improve the accuracy of runoff forecasting. However, this paper only applies this model to the daily runoff prediction at four hydrological stations in the lower reaches of the Yellow River, and the model can be applied to different hydrological stations and different time scales to explore the applicability of this method.
4. The kernel-limit learning machine model proposed in this paper via variational modal decomposition and ensemble empirical modal decomposition with the butterfly optimisation algorithm still has some limitations. If the input data contains outliers or noise, the performance of the model may be severely affected, which requires preprocessing of the data and outlier detection to ensure the robustness of the model.

## Data Availability

Data and materials are available from the corresponding author upon request.

## References

[1] Chiew FHS, Young WJ, Cai W, Teng J (2011). Current drought and future hydroclimate projections in southeast Australia and implications for water resources management. Stoch. Env. Res. Risk Assess..

[2] Medina Y, Muñoz E (2020). Analysis of the relative importance of model parameters in watersheds with different hydrological regimes. Water.

[3] Horuz CC, Karlbauer M, Praditia T, Butz MV, Oladyshkin S, Nowak W, Otte S (2023). Physical domain reconstruction with finite volume neural networks. Appl. Artif Intell..

[4] Xiong P, Zou X, Yang Y (2021). The nonlinear time lag multivariable grey prediction model based on interval grey numbers and its application. Nat. Hazard..

[5] Zhang G, Sheng Y, Shi Y (2022). Uncertain hypothesis testing of multivariate uncertain regression model. J. Intell. Fuzzy Syst..

[6] Rahman, M. S., Khomh, F., Hamidi, A., Cheng, J., Antoniol, G., & Washizaki, H. Machine learning application development: practitioners’ insights. Softw. Qual. J., 1–55. (2023).

[7] Li Q, Liu Y, Wang S, Gao Q, Gao X (2018). Image classification using low-rank regularized extreme learning machine. IEEE Access.

[8] Qiao X, Peng T, Sun N, Zhang C, Liu Q, Zhang Y, Nazir MS (2023). Metaheuristic evolutionary deep learning model based on temporal convolutional network, improved aquila optimizer and random forest for rainfall-runoff simulation and multi-step runoff prediction. Expert Syst. Appl..

[9] Huang S, Yu L, Luo W, Pan H, Li Y, Zou Z, Chen J (2023). Runoff prediction of irrigated paddy areas in Southern China based on EEMD-LSTM model. Water.

[10] Wang WC, Wang B, Chau KW, Xu DM (2023). Monthly runoff time series interval prediction based on WOA-VMD-LSTM using non-parametric kernel density estimation. Earth Sci. Inf..

[11] Lian L (2022). Runoff forecasting model based on CEEMD and combination model: a case study in the Manasi River, China. Water Supply.

[12] Zhang X, Tuo W, Song C (2020). Application of MEEMD-ARIMA combining model for annual runoff prediction in the Lower Yellow River. J. Water Clim. Change.

[13] Yan X, Chang Y, Yang Y, Liu X (2021). Monthly runoff prediction using modified CEEMD-based weighted integrated model. J. Water Clim. Change.

[14] Lu H, Du B, Liu J, Xia H, Yeap WK (2017). A kernel extreme learning machine algorithm based on improved particle swam optimization. Memet. Comput..

[15] Song C, Yao L, Hua C, Ni Q (2021). Comprehensive water quality evaluation based on kernel extreme learning machine optimized with the sparrow search algorithm in Luoyang River Basin, China. Environ. Earth Sci..

[16] Wang Z, Wang Q, Wu T (2023). A novel hybrid model for water quality prediction based on VMD and IGOA optimized for LSTM. Front. Environ. Sci. Eng..

[17] Yang H, Li W (2023). Data decomposition, seasonal adjustment method and machine learning combined for runoff prediction: A case study. Water Resour. Manag..

[18] Huang S, Chang J, Huang Q, Chen Y (2014). Monthly streamflow prediction using modified EMD-based support vector machine. J. Hydrol..

[19] Wu Z, Huang NE (2009). Ensemble empirical mode decomposition: a noise-assisted data analysis method. Adv. Adapt. Data Anal..

[20] Kim HJ, Kim C, Choi Y, Wang S, Zhang X (2010). Improved modification direction methods. Comput. Math. Appl..

[21] Zheng Y, Chen B, Wang S, Wang W, Qin W (2020). Mixture correntropy-based kernel extreme learning machines. IEEE Trans. Neural Netw. Learn. Syst..

[22] Huang GB (2014). An insight into extreme learning machines: Random neurons, random features and kernels. Cogn. Comput..

[23] Aljafari B, Balachandran PK, Samithas D, Thanikanti SB (2023). Solar photovoltaic converter controller using opposition-based reinforcement learning with butterfly optimization algorithm under partial shading conditions. Environ. Sci. Pollut. Res..

[24] Yu, N., Yang, X., Feng, R., & Wu, Y. (2023). Strain signal denoising based on adaptive variation mode decomposition (VMD) algorithm. J. Low Freq. Noise Vib. Active Control, 14613484231187773.

[25] Ayana Ö, Kanbak DF, Kaya Keleş M, Turhan E (2023). Monthly streamflow prediction and performance comparison of machine learning and deep learning methods. Acta Geophys..

